# Point-of-Care Ultrasound for the Diagnosis of Frequent Cardiovascular Diseases: A Review

**DOI:** 10.7759/cureus.51032

**Published:** 2023-12-24

**Authors:** Ernesto Calderon Martinez, Edna Diarte, Diana Othon Martinez, Leonardo Rodriguez Reyes, Daniel A Aguirre Cano, Carolina Cantu Navarro, Maria G Ycaza Zurita, David Arriaga Escamilla, Jinal Choudhari, George Michel

**Affiliations:** 1 Digital Health, Universidad Nacional Autónoma de México, Ciudad de Mexico, MEX; 2 Medicine, Universidad Autónoma de Sinaloa, Culiacán, MEX; 3 Internal Medicine, University of Texas Rio Grande Valley, Edinburg, USA; 4 Internal Medicine, Universidad de Monterrey, Monterrey, MEX; 5 Internal Medicine, Universidad Anahuac, Monterrey, MEX; 6 Internal Medicine, Columbia University, Bronx, USA; 7 Internal Medicine, Universidad Justo Sierra, Mexico City, MEX; 8 Research & Academic Affairs, Larkin Community Hospital, South Miami, USA; 9 Internal Medicine, Larkin Community Hospital, South Miami, USA

**Keywords:** cardiovascular assessment, cardiology, ultrasound, point-of-care, pocus

## Abstract

Point-of-care ultrasound (POCUS) has emerged as an indispensable diagnostic tool in cardiology, particularly within the emergency department. This narrative synthesis provides a comprehensive exploration of POCUS applications in cardiovascular diseases, elucidating its multifaceted roles and addressing challenges. The review delves into the technical attributes of POCUS, emphasizing its non-invasive nature, radiation-free qualities, and suitability for non-radiologists. It navigates through educational strategies, stressing the importance of structured programs for the seamless integration of POCUS into clinical practice. Highlighting its efficacy, the synthesis discusses POCUS applications in various scenarios such as dyspnea, chest pain, cardiac arrest, aortic dissection, pericardial effusion, and pulmonary embolism. Beyond acute care, the review explores the role of POCUS in outpatient and inpatient settings, focusing on chronic and acute heart failure, valvular heart diseases, and more. Acknowledging operator-dependent challenges and the need for continuous education, the review underscores the transformative potential of POCUS across diverse healthcare settings. This narrative synthesis accentuates POCUS as a valuable and versatile diagnostic tool in cardiology, offering efficiency, safety, and cost-effectiveness. Despite challenges, POCUS stands out as a transformative addition to clinical practices, poised to enhance patient outcomes and reshape the landscape of cardiovascular diagnostics.

## Introduction and background

The emergency department (ED) receives patients with diagnoses ranging from cardiovascular etiology to trauma and lung disease [1-3]. Finding a standardized test to aid in diagnostic results might be difficult. Some studies suggest that there is evidence that the use of point-of-care ultrasound (POCUS) is beneficial for assessing patients in the ED [4]. Additionally, they suggest that its incorporation into the Advanced Trauma Life Support (ATLS) algorithm and other trauma algorithms may lead to more rapid diagnosis and better disease outcomes [5,6]. POCUS is performed on the bedside, allowing a more thorough physical examination and aiding in a more accurate diagnosis.

From all the aspects of POCUS, lack of radiation, accessibility, and decrease in time to reach a diagnosis are among the most popular benefits of this modality [7-9]. POCUS can be operated by non-radiologists [4]. This has increased interest among residents and other healthcare providers significantly over the last few years, making the training accessible [10,11].

This paper aims to provide pertinent technical information and identify obstacles to the successful integration and impact of POCUS, highlighting the benefits and limitations of these devices and demonstrating the many capabilities of POCUS in the ED, outpatient, and inpatient setting to highlight the importance of this new tool in patient care. Additional information discussing POCUS implementation on the diagnosis of cardiovascular disease and its current use in the medical field is also included.

## Review

Methods

This review synthesizes existing research on POCUS and its use in common cardiovascular diseases. Literature from various sources (PubMed, Scopus, Medline, and Google Scholar) was systematically searched and reviewed. Studies included in this review encompassed a diverse cohort of cardiovascular patients, covering different age groups and diseases. The selection criteria included studies using POCUS as a diagnostic tool and patients with cardiovascular disease.

POCUS

POCUS is defined as ultrasonography brought to the patient and performed by the provider in real-time, allowing findings to be directly correlated with the patient’s presenting signs and symptoms and can be easily repeatable if the patient’s condition changes [12].

Platforms and devices

**Figure 1:**
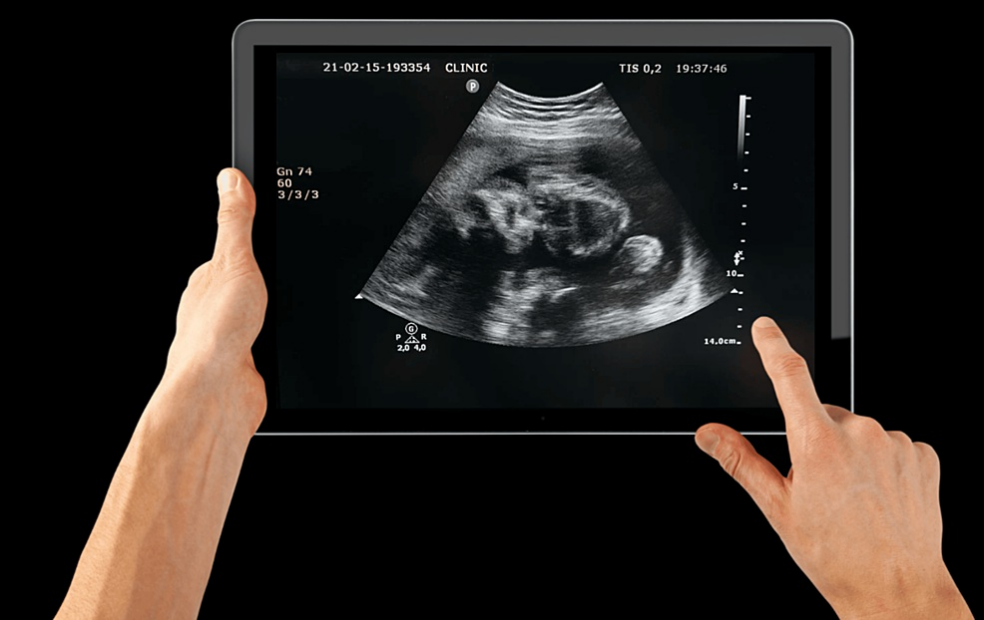
Example of a display application on an iPad (Apple Inc., Cupertino, CA) Image credits: Calderon Martinez Ernesto.

All the POCUS devices are non-invasive, low-risk imaging modalities that can be used to diagnose and help guide the management of critically ill adults and children in the emergency room in the cardiac intensive care unit (ICU) [13]. The devices provide rapid real-time images that can be performed at the bedside in patients in the emergency department when suspecting cardiac dysfunction and in the pediatric cardiac ICU performed by the intensivist [14]. Generally speaking, POCUS is a compact device designed for use in limited spaces or situations where traditional examinations may be impractical. Its aim is to facilitate swift clinical decision-making, fostering the potential for enhanced patient outcomes. POCUS devices have significantly improved, and the image quality is reasonably good when utilized by a trained provider, though it may still be limited by body habitus. Harmonic imaging is a feature of many systems. Color flow Doppler is widely available. Spectral Doppler (pulsed or continuous wave) is available on some systems. Other systems have implemented measurement packages and applications. Most systems now allow storage in DICOM (Digital Imaging and Communications in Medicine) format to allow uploading to picture archiving and communication systems (PACS). Wireless and Bluetooth technology now facilitate transducer recognition, battery charging, and image transfer. Touchscreen technology is common, and screen sizes have become so small that they either fit in a pocket or utilize a display application on a cell phone (Figure 1).

Unique probe technology that uses a silicon chip array instead of piezoelectric crystals has become available, allowing images to be displayed in various formats that would have previously required separate probes. Lastly, artificial intelligence has crept into the POCUS world, using technology-assisted image acquisition for less experienced users [15,16].

POCUS is a valuable tool in clinical practice, but it does have inherent limitations in diagnosing common clinical issues like ischemic heart disease or pulmonary embolism. POCUS may not always provide a comprehensive view of the entire organ or vasculature, potentially missing subtle or distal abnormalities, and the diagnostic accuracy is always below normal ultrasound. Additionally, operator expertise plays a significant role in the accuracy of POCUS diagnosis, and it may not replace more advanced imaging modalities for definitive assessments in complex cases. Therefore, while POCUS is a valuable initial assessment tool, its limitations should be considered when evaluating patients with these conditions [15,17].

Education

Some studies showed that the most common barriers to using POCUS in decision-making are the need for more trained providers and the inability to use results in the documentation. They also showed a strong desire for POCUS training and their perception of the importance of skill learning and practice [10,11]. It is important that the training on POCUS needs have a correct structure according to the level of the student, and the trainer needs to have continued academic and teaching training to have the best approach [18]. Several studies showed excellent performance in the students and residents of medicine who had at least two-hour courses or more in POCUS, showing that they can perform cardiovascular studies with high sensitivity and specificity despite the fact they are students or advanced medical trained [19-21].

The American Society of Echocardiography (ASE) recently released recommendations to guide accredited echocardiography laboratories in developing cardiac POCUS training programs for non-cardiologists. They differ in their level of training (medical student, resident, or attending physician) and, consequently, prior knowledge of ultrasound [15].

Effectiveness

Some studies have shown that the use of POCUS increases the percentage of adequate diagnosis, decreases the time to diagnosis, abnormalities reported, and decreases the rate of patients triage to the ICU, the number of interventions, use of mechanical ventilation, delay in treatment, systemic steroids, and vasopressors, without significant change on the perioperative patient [22,23]. Also, when used correctly, POCUS increases the physician's confidence and decreases the workload, mental demand, and frustration level [24,25].

Imaging modalities

The ED is where time and efficient decision-making are essential for the patient’s well-being. Physician’s clinical experience and diagnostic support tools directly impact disease outcomes. Among the symptoms and diagnoses that lead to ED visits, chest pain and cardiac etiologies are among the most common; diagnostic tools such as transesophageal echocardiography (TTE) and computed tomography (CT) are most commonly used and cannot be substituted by POCUS. POCUS remains as a confirmatory tool or as a tool to suspect the disease, always trying to achieve the accuracy of TTE [19]. Thoracic trauma leading to pneumothorax accounts for many traumatic deaths in the ED, with chest X-ray (CXR) used as an initial assessment [20].

These days, the ATLS guidelines include the POCUS as part of the Focused Assessment with Sonography in Trauma (FAST) assessment due to the high sensibility for diagnosing trauma injuries that threaten life [26,27].

CT remains a gold standard for multiple diagnoses, cardiac and non-cardiac. It is particularly useful in identifying perfusion defects [28]. TTE without or with contrast (cTTE) allows morphology assessment and functionality. Ideal for coronary artery disease, structural heart disease evaluation, and ischemic stroke. On the other hand, CT and TTE interpretation and implementation require specific training [28,29].

POCUS in the diagnosis of common heart diseases

Cardiac diseases are the leading cause of death globally, with over 17.9 million deaths attributed to them in 2016 alone [1]. Due to the great economic burden on health systems, more efficient and less costly diagnostic methods have been sought. Among these tools is POCUS, which has gained much importance in cardiology [2,3].

Emergency

POCUS complements the diagnostic skills of the ED physicians improving and accelerating various differential diagnoses by approximately 20 minutes than traditional ED evaluation when more advanced tools are not available [7-9].

Dyspnea

All scans (of the heart and lung) can be valuable in ruling in/out other possible differential diagnoses for dyspnea [30]. Dyspnea is a common subjective symptom that can be present in several pathologies involving the heart and lungs. POCUS can be utilized in the work-up for diagnostic evaluation, it is usually readily available and can be utilized by physicians in training. Patients can be evaluated in their bed, in different positions, avoiding discomfort. Findings, including the B-line pattern, are highly suggestive of pulmonary edema [31]. Even if these findings cannot elucidate a clear diagnosis, some studies describe cardiogenic B lines as regular and B-lines from pneumonia or acute respiratory distress syndrome as irregular [31]. The bedside lung ultrasonography in emergency (BLUE) protocol helps physicians to differentiate the reason for dyspnea between thrombosis, pulmonary edema, pneumonia, or acute respiratory distress syndrome, helping in the decision-making process [3,7].

Chest pain

POCUS is better known and more common in the context of emergency and ICU. As dyspnea and chest pain are two of the most common symptoms in the ED, POCUS can be very useful in shortening the possible differential diagnoses. It may not allow the physician to immediately rule out cardiovascular reasons, but it could identify other possible causes of the symptom, ranging from respiratory to cardiovascular to abdominal disorders, and help in the decision-making process [3,7].

Cardiac arrest

Recently, POCUS has been used to identify early correctable causes of cardiac arrest. We have sought to systematize the use of POCUS in this clinical context. These advantages lie in the real-time assessment of potentially reversible causes of cardiorespiratory arrest. Nevertheless, the information currently available has some limitations, as it only reports lower cohorts and has a high risk of bias; there is a need for further research on this topic that could help in the decision-making process in real time [32].

Aortic dissection, pericardial effusion, and pulmonary embolism

POCUS has been demonstrated to significantly reduce the time to diagnosis of these pathologies in different studies, with high specificity and sensibility to diagnose the disease, and reduction on mortality and mortality on follow-up, despite these promising findings, there remains a crucial need for additional evidence. It is worth noting that standard transesophageal echocardiogram, while valuable, exhibits reduced sensitivity in diagnosing aortic dissection and pulmonary embolism, and that POCUS is recommended when there are no advanced tools to diagnose the patients, helping the ED physicians in the decision-making process [33-37].

Outpatient/inpatient

Although its role in non-emergency settings is still being studied, it is known that it can be helpful in physical examination [38]. Also, POCUS has been demonstrated to be a good substitute for traditional general clinical examination when used by trained hospitalists, residents, or medical students for decision-making [39,40].

Chronic and acute heart failure

Heart failure has also increased in prevalence in recent years; an estimated 64.3 million people are living with heart failure worldwide; in developed countries, the prevalence of heart failure is 1% to 2% in the adult population [41]. The finding of a congested inferior vena cava would suggest this diagnosis [42]. For acute decompensated heart failure (ADHF), it is necessary to use a method with high diagnostic accuracy. Additionally, serial POCUS examinations have been described to improve the therapeutic approach and decrease the time of patient-reported symptom relief in patients with acute exacerbation of heart failure, likely due to a strict therapeutic diuretic approach, based on imaging findings [43].

Valvular heart diseases

Another cardiovascular disease that affects a great proportion of the human population is valvular cardiac disease. The most affected valves of the heart are the aortic valve and the mitral valve, these affections are consequences of rheumatic heart disease (RHD) that could lead mainly to mitral stenosis, which normally occurs in children or young adults [44]. Studies have shown that POCUS is superior to conventional auscultation for diagnosing valvular heart disease, even when performed by non-cardiologists. Examination with POCUS has been shown to improve the diagnostic accuracy of valvular heart disease by 50% to 80% in as little as 15 minutes after the start of the physical examination, even in final-year medical students increasing their percentage of valvular heart disease detection [45].

Arrhythmias

Arrhythmias are also common cardiac diseases. Atrial fibrillation (AF) is the heart's most common type of irregular heart rhythm [46]. POCUS has been studied as the initial approach to arrhythmias in pediatrics, as it is less invasive and costly than other methods, but this use requires the training of healthcare providers and is still in debate [42]. However, its usefulness in different arrhythmias needs to be further studied to conclude, so it represents an important area for further research.

Pediatric

POCUS can be a valuable tool in diagnosing and managing pediatric cardiac diseases. It has represented a transformative change in clinical practice by displacing the classical stethoscope [47]. It allows healthcare providers to obtain real-time imaging of the heart's surrounding structure at the bedside, aiding in rapid assessment and decision-making [47,48].

One of the most common applications of POCUS is heart failure, which allows healthcare providers to obtain real-time imaging of cardiac activity and pericardial effusion while assessing cardiac function [49]. It plays a role in the early recognition of acute heart failure, both in children and in the adult population.

Finally, the use of POCUS in the emergency room is a useful modality for evaluating cardiac function and confirming the diagnosis of myocarditis in the pediatric population with COVID-19 [48], a disease that has been increasing in this specific population due to the still unknown short or long complication for this virus [50].

POCUS use and implementation have expanded significantly over the last decade, and it may now be referred to as the 21st-century stethoscope. POCUS applications in medical diagnosis are progressively expanding in almost all medical specialties. Unfortunately, the quality of ultrasound examination can be affected by the physician's experience and the patient's body weight. There is a need to establish a unified, integrated formal curriculum and adequate training to safely and effectively use POCUS [51-53]. Considering all its diagnostic advantages (Table 1), in addition to its effectiveness and safety, it can be concluded that POCUS is a very useful diagnostic aid in cardiology.

**Table 1:** Evidence of the use of POCUS in inpatient and emergency room settings POCUS: point-of-care ultrasound; IVC: inferior vena cava.

Authors	Setting	Study	Year	Country	Sample size	Disease	Comment
Zanobetti et al. [8]	Emergency room	Prospective	2017	Italy	2,683 patients	Acute dyspnea	The average time needed to diagnose was 24 ± 10 minutes in the POCUS group vs. 186 ± 72 minutes in the control group. There were no significant accuracy differences between POCUS and the standard evaluation for the diagnosis.
Alpert et al. [35]	Emergency room	Retrospective cohort study	2017	Israel	73 patients	Cardiac tamponade	The door-to-pericardiocentesis time was decreased, i.e., 11.3 hours in the POCUS group vs. 70.2 hours in the control group. Also, the length of hospital stay was shorter by 2 days in the intervention group, 5.1 days in the POCUS group vs. 7.0 days in the control group.
Hanson et al. [37]	Emergency room	Retrospective	2021	Canada	342 patients	Cardiac tamponade	The door-to-diagnosis with POCUS and departmental echo were on average 5.9 hours and 45.1 hours, respectively. The door-to-pericardiocentesis time was 28.1 hours in the POCUS group in comparison to 49 hours for the departmental echo group.
Núñez-Ramos et al. [43]	Inpatient	Retrospective cohort	2023	Colombia	149 patients	Heart failure	The median time to diuretic treatment was 78 minutes (25-578 minutes) in the global population. In the clinical group compared to the POCUS group, the median time to disposition decision was 360 minutes (180–545 minutes) versus 235 minutes (95.5-410 minutes). The median global length of hospital stay was 6 days in the clinical group compared to 3 days in the POCUS group.
Sobczyk and Nycz [54]	Inpatient	Retrospective	2015	Poland	178 patients	Aortic dissection	Statistical analysis with the chi-square test did not show any statistically significant differences between computed tomography and echocardiography in the detection of the proximal aortic dissection. Additionally, bedside transthoracic echocardiography revealed concomitant abnormalities such as bicuspid aortic valve, atrioventricular calcifications, moderate/severe aortic incompetence, or cardiac tamponade.
Lu et al. [55]	Inpatient	Retrospective observational	2020	USA	141 patients	Hemodynamic instability	Cardiac pathology was positive in 129 (68%) of the rescue examinations. Common reported pathologies included left ventricular dysfunction (25%), right ventricular dysfunction (28%), hypervolemia (7%), hypovolemia (13%), and pericardial effusion/tamponade (11%). Seventy-five percent of the rescue examinations resulted in interventions, including fluid resuscitation (13%), diuresis (7%), ionotropic support (12%), surgical intervention in the operating room (11%), surgical intervention at the bedside (4%), and extracorporeal membrane oxygenation initiation (8%).
Wang et al. [56]	Emergency room	Prospective	2020	China	127 patients	Aortic dissection	Compared with computed tomographic angiography, the sensitivity of POCUS was 86.4%, and the specificity was 100.0%. The door-to-diagnosis times were 10.5 minutes in the group where POCUS was used and 79.0 minutes in the control group.
Nazerian et al. [57]	Emergency room	Prospective	2014	Italy	281 patients	Aortic dissection	Detection of any focused cardiac ultrasound sign (direct or indirect) of aortic disease had a sensitivity of 88% and specificity of 94%, combination of aortic disease score of >1 point and ultrasound has a 98%.
Pare et al. [58]	Emergency room	Retrospective	2016	USA	32 patients	Aortic dissection	The door-to-diagnosis time in the ultrasound group was 80 minutes and 226 minutes in the non-ultrasound group. The misdiagnosis rate was 0% in the ultrasound group and 43.8% in the non-ultrasound group. The mortality percentage was 15.4% in the non-ultrasound group vs. 37.5% in the ultrasound group, which was not statistically significant (P = 0.24).
Nazerian et al. [59]	Emergency room	Prospective	2015	Italy	140 patients	Ascending aorta dilation and aneurysm	Ascending aorta dilation and aneurysm were detected with ultrasound in 35.7% and 17.8% of patients, respectively. The ultrasound had 78.6% sensitivity and 92.9% specificity for ascending aorta dilation compared with computed tomographic angiography. The ultrasound had a sensibility of 64.7% and specificity of 95.3% for ascending aorta aneurysm compared with computed tomographic angiography.
Taylor et al. [60]	Emergency room	Retrospective	2012	USA	92 patients	Ascending aorta dilation and aneurysm	The ultrasound had 77% sensitivity and 95% specificity for ascending aorta dilation. The ultrasound had a sensibility of 64% and specificity of 90% for ascending aorta aneurysm.
Hoch et al. [61]	Emergency room	Retrospective cohort study	2022	USA	257 patients	Cardiac tamponade	The door-to-pericardiocentesis time was decreased, i.e., 21.6 hours in the POCUS group compared to 34.6 hours in the No POCUS group. Pericardial effusion was associated with a decreased 28-day mortality of 9.7% in the POCUS group vs. 26.0% in the non-POCUS group.
Milne et al. [62]	Emergency room	Multicenter prospective	2016	Canada	150 patients	Cardiac arrest and undifferentiated hypotension	In undifferentiated hypotension, POCUS findings were left ventricular dynamic changes (43%), IVC abnormalities (27%), pericardial effusion (16%), and pleural fluid (8%). In cardiac arrest, POCUS findings were abnormalities of ventricular contraction (45%) and valvular motion (39%).
Huang et al. [63]	Emergency room	Prospective	2023	Taiwan	465 patients	Chest pain/dyspnea	The use of POCUS was associated with shorter length of stay and patient survival.
Lichtenstein et al. [64]	ICU	Observational	2008	France	260	Pulmonary edema	The characteristic B line pattern was observed in cardiogenic pulmonary edema. It was calculated with a sensitivity of 97% and a specificity of 95%. Diffuse B+ lines were also observed in patients with pneumonia, it was calculated with a sensitivity of 11% and specificity of 100%.
Prosen et al. [65]	Emergency room	Prospective	2011	Slovenia	218	Pulmonary edema	The ultrasound comet-tail sign has 100% sensitivity, 95% specificity, 100% negative predictive value, and 96% positive predictive value for the diagnosis of heart failure.

Limitations in the application of POCUS in the diagnosis and management of common cardiovascular conditions

While POCUS is considered a valuable tool for diagnosing and managing common cardiovascular conditions, early discussions at the inaugural international conference on focused cardiac ultrasound (FoCUS) highlighted that the reliability of cardiac ultrasound in everyday clinical practice heavily depends on the operator's perception, especially when utilized by clinicians not specialized in cardiology. This operator-dependent factor can result in divergent or inconclusive outcomes, largely influenced by the practitioner's skills developed during training and experience [66-69]. Moreover, the persistent challenge of maintaining ongoing education among healthcare professionals and ensuring the effective implementation of quality assurance measures post-training is magnified [70-72]. Even seasoned practitioners could face difficulties in challenging clinical settings due to the qualitative reporting nature or when assessing subtle or complex abnormalities [71,73]. In low-income countries, the limited accessibility and resources create barriers to POCUS adoption, impacting diagnosis accuracy and training [74,75]. The call for standardization was echoed in developing the Study of Heart and Renal Protection (SHARP) protocol, which demonstrated that standardized procedures are essential for reliable assessment. POCUS consistently yielded good to excellent image quality facilitating [76]. The utility of handheld POCUS, while promising, may encounter substantial challenges when striving for precise diagnoses and effective management of cardiovascular conditions, particularly in the presence of concurrent comorbidities [77]. Another challenge is the patient perspective, there is evidence that patients report a high percentage of need for clearer communication and patient education regarding the scope and limitations of POCUS. Nevertheless, most patients in the study reported positive experiences with POCUS. Most patients reported a substantial percentage of being taken more seriously and thoroughly examined, a better understanding of their health problem, more security, and increased trust in the physician assessment [78].

Patient approach

A cross-sectional study on the Danish population showed a high percentage of patients well informed about the process and result of POCUS use. Also, a significant portion reported naturally integrated into these devices consultation. In addition, most patients in this study reported a substantial percentage of being taken more seriously and thoroughly examined, a better understanding of their health problem, more security, and increased trust in the physician assessment. All this demonstrated and reported an improvement in the doctor-patient relationship and improvement of service and quality care [79].

## Conclusions

In summary, this review underscores the versatile role of POCUS in diagnosing common cardiovascular diseases. POCUS provides a valuable, non-invasive diagnostic tool in emergency and inpatient settings. The technological advancements in POCUS devices, including wireless capabilities and artificial intelligence integration, enhance its convenience and diagnostic potential. Despite its advantages, we acknowledge the limitations, particularly in complex cases. Operator expertise is crucial, emphasizing the need for standardized training. Studies show that POCUS improves diagnostic speed, accuracy, and patient outcomes in scenarios like dyspnea, chest pain, and cardiac arrest. In outpatient and inpatient settings, POCUS aids in chronic and acute heart failure and valvular heart diseases, among others. Evidence supports its efficacy in diagnosing aortic dissection, pericardial effusion, and pulmonary embolism, contributing to timely interventions. Challenges in widespread POCUS implementation include operator-dependent factors, ongoing education needs, and resource limitations. Patient perspectives highlight positive experiences, emphasizing improved doctor-patient relationships and increased trust in assessments. In conclusion, POCUS serves as a transformative diagnostic aid in cardiology, offering efficiency and improved patient outcomes. Standardization and ongoing training are crucial for maximizing its potential, representing a significant advancement in clinical practice.
